# Supplementary material for: Progressive changes in coral reef communities with increasing ocean acidification
Source: Commun Biol. 2025 Nov 24;8:1518. doi: 10.1038/s42003-025-08889-w (PMC12644485; doi:10.1038/s42003-025-08889-w)
Supplement: Supplementary file 2 — Supplementary information [file 42003_2025_8889_MOESM2_ESM.pdf]

Supplementary information for Noonan *et al.*

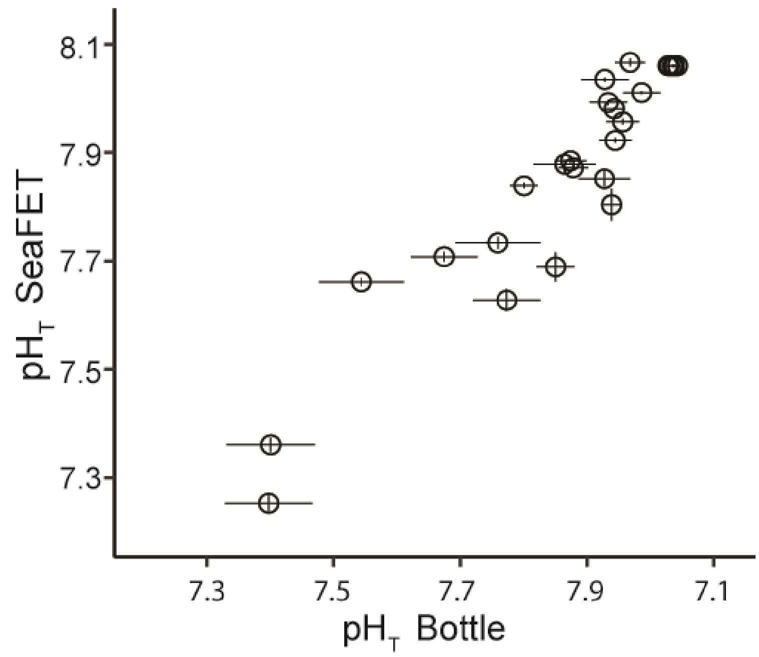

Supplementary Fig. 1: The mean pH_{Total} ± SE at each sampling station along the gradient of seep exposure as measured from bottle samples and with the SeaFET loggers. Error bars are standard errors.

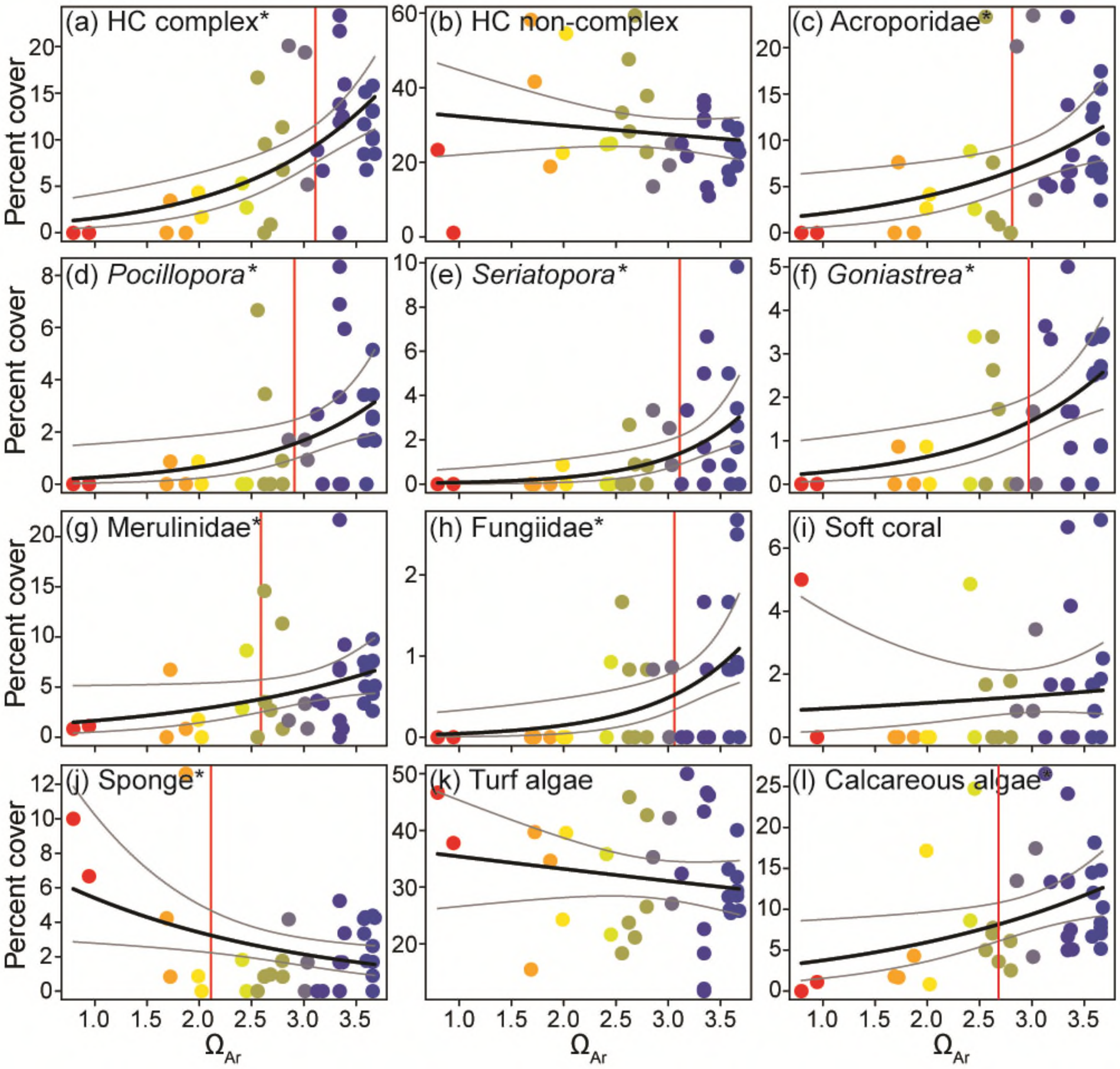

Supplementary Fig. 2: The percent cover of all complex hard corals (HC) (a), non-complex hard corals (b), Acroporidae (c), *Pocillopora* spp. (d), *Seriatopora* spp. (e), *Goniastrea* spp. (f), Merulinidae (g), Fungiidae (h), Soft corals (i), Sponges (j), turf algae (k), and all calcareous algae (l) in relation to mean aragonite saturation state (Ω_{Ar}). Point colour represents station mean Ω_{Ar} (legend as per Fig. 2). The black line is the modelled mean, and the grey lines are 95% confidence intervals. The red vertical line is the no-significant-effect concentrations (NSEC). * denotes statistical significance in generalised linear models at $p < 0.05$ (Table S2).

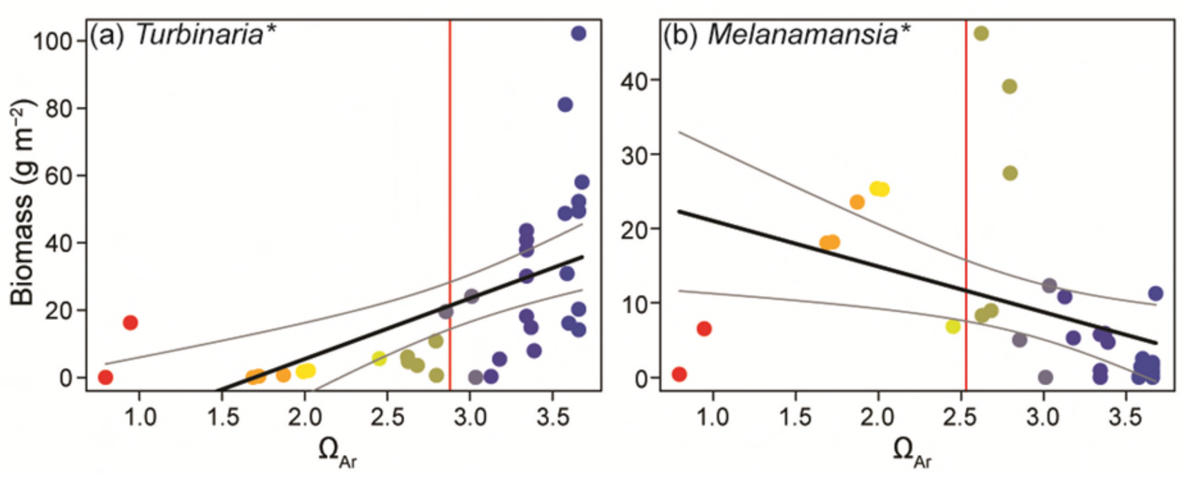

Supplementary Fig. 3: The biomass of the abundant macroalgal genera *Turbinaria* (a) and *Melanamansia* spp. (b) in relation to mean aragonite saturation state (Ω_{Ar}). Point colour represents station mean Ω_{Ar} (legend as per Fig. 2). The black line is the modelled mean, and the grey lines are 95% confidence intervals. The red vertical line is the no-significant-effect concentrations (NSEC). * denotes statistical significance in generalised linear models at $p < 0.05$ (Table S2).

Supplementary Table 1: Temperature (°C) and carbon chemistry parameters from the stations at the volcanic seep at Upa Upasina, Normamby Island, PNG. Measured values of pH_{Total} (combined bottle and SeaFET measurements) and total alkalinity (A_T: µmol kg^{-1}), and calculated pCO_2 (µatm), total dissolved inorganic carbon (C_T: µmol kg^{-1}), bicarbonate (HCO_3^-: µmol kg^{-1}), carbonate (CO_3^{2-}: µmol kg^{-1}), and the saturation states of aragonite (Ω_{Ar}) and calcite (Ω_{Ca}) at the ambient sampling stations unaffected by the volcanic CO_2 and those within the seep. Mean values are reported (± SE).

Site	Stations	Temp	pH_T	A_T	pCO_2	C_T	HCO_3^-	CO_3^{2-}	Ω_{Ar}	Ω_{Ca}
Ambient CO_2	1	28.45 (0.01)	8.06 (<0.01)	2228 (12)	369 (2)	1906 (11)	1670 (9)	227 (1)	3.69 (0.02)	5.54 (0.03)
	2		8.04 (<0.01)	2234 (9)	389 (1)	1923 (8)	1692 (7)	220 (1)	3.58 (0.02)	5.38 (0.02)
	3		8.04 (<0.01)	2251 (6)	393 (1)	1938 (6)	1706 (5)	222 (1)	3.60 (0.01)	5.42 (0.02)
	4		8.05 (<0.01)	2253 (5)	397 (1)	1942 (5)	1710 (4)	221 (1)	3.59 (0.01)	5.40 (0.01)
	5		8.04 (<0.01)	2252 (5)	398 (1)	1941 (5)	1710 (4)	220 (1)	3.58 (0.01)	5.38 (0.01)
	6	27.98 (<0.01)	8.00 (0.01)	2263 (8)	450 (2)	1976 (7)	1758 (7)	206 (1)	3.34 (0.01)	5.03 (0.02)
Seep	1	27.96 (<0.01)	8.02 (0.01)	2215 (12)	415 (2)	1921 (11)	1701 (10)	209 (1)	3.39 (0.02)	5.10 (0.03)
	2	28.04 (0.01)	8.00 (0.01)	2251 (5)	438 (1)	1960 (5)	1741 (4)	208 (<1)	3.37 (0.01)	5.07 (0.01)
	3	27.95 (0.01)	7.97 (0.01)	2255 (8)	482 (2)	1983 (8)	1774 (7)	196 (1)	3.18 (0.01)	4.79 (0.02)
	4	27.47 (0.02)	7.96 (<0.01)	2257 (9)	496 (2)	1990 (9)	1784 (8)	193 (1)	3.13 (0.01)	4.71 (0.02)

5	27.53 (0.02)	7.95 (0.01)	2236 (2)	512 (1)	1979 (2)	1780 (2)	186 (<1)	3.01 (<0.01)	4.53 (<0.01)
6	NA	7.94 (0.02)	2259 (8)	519 (2)	2001 (8)	1800 (7)	187 (1)	3.03 (0.01)	4.57 (0.02)
7	28.16 (0.04)	7.92 (0.01)	2239 (13)	556 (3)	1997 (12)	1807 (11)	176 (1)	2.85 (0.02)	4.30 (0.03)
8	NA	7.90 (0.03)	2257 (23)	585 (7)	2022 (22)	1835 (20)	172 (2)	2.80 (0.03)	4.21 (0.04)
9	NA	7.90 (0.02)	2270 (7)	595 (2)	2036 (7)	1848 (6)	172 (1)	2.79 (0.01)	4.20 (0.01)
10	27.90 (0.02)	7.87 (0.01)	2277 (19)	637 (5)	2055 (18)	1873 (16)	165 (1)	2.68 (0.02)	4.04 (0.04)
11	27.97 (0.02)	7.87 (0.02)	2264 (14)	645 (4)	2046 (13)	1867 (12)	162 (1)	2.63 (0.01)	3.96 (0.03
12	27.97 (0.04)	7.86 (0.01)	2261 (9)	646 (2.71)	2044 (9)	1865 (8)	162 (1)	2.62 (0.01)	3.95 (0.02)
13	28.08 (0.04)	7.86 (0.02)	2241 (3.05)	654 (1)	2030 (3)	1855 (3)	158 (<1)	2.56 (<0.01)	3.85 (<0.01)
14	27.98 (0.11)	7.83 (0.02)	2258 (10)	708 (3)	2058 (9)	1888 (8)	151 (1)	2.45 (0.01)	3.69 (0.02)
15	27.99 (0.01)	7.82 (0.01)	2260 (8)	726 (3)	2064 (8)	1896 (7)	149 (1)	2.41 (0.01)	3.63 (0.01)
16	28.09 (0.01)	7.73 (0.02)	2274 (28)	934 (12)	2119 (27)	1970 (25)	125 (2)	2.02 (0.03)	3.04 (0.04)
17	27.85 (<0.01)	7.72 (0.03)	2254 (9)	933 (4)	2101 (9)	1954 (8)	123 (1)	1.99 (0.01)	3.00 (0.01)
18	28.02 (0.01)	7.69 (0.02)	2269 (15)	1027 (7)	2130 (14)	1987 (13)	115 (1)	1.87 (0.01)	2.82 (0.02)

19	28.53 (0.02)	7.65 (0.03)	2269 (11)	1141 (6)	2146 (11)	2010 (10)	106 (<1)	1.72 (0.01)	2.59 (0.01)
20	28.02 (0.02)	7.63 (0.02)	2328 (25)	1243 (14)	2212 (24)	2076 (23)	104 (1)	1.69 (0.02)	2.54 (0.03)
21	27.86 (<0.01)	7.36 (0.03)	2297 (30)	2396 (32)	2276 (30)	2155 (29)	58 (1)	0.94 (0.01)	1.41 (0.02)
22	28.76 (0.02)	7.27 (0.03)	2331 (23)	2998 (30)	2340 (24)	2212 (22)	50 (<1)	0.79 (0.01)	1.20 (0.01)

Supplementary Table 2: Generalised linear model results for the different benthic community characteristics (cover, abundances, diversity and biomass) in response to the aragonite saturation state gradient (Ω_{Ar}). Data were modelled to quasibinomial (^{qb}), quasipoisson (^{qp}) and gaussian (^{g}) distributions. Coefficient estimates are reported in the scale of the link function. Some data are square root transformed ($^{0.25}$) and bold p-values indicate statistical significance at $p < 0.05$.

	Estimate	SE	t	P
**Percent cover**				
**HC** ^{qb}				
Intercept	-0.89	0.35	-2.57	**0.014**
Ω_{Ar}	0.12	0.12	1.06	0.290
**Massive *Porites* spp.** ^{qb}				
Intercept	-0.78	0.49	-1.58	0.122
Ω_{Ar}	-0.27	0.17	-1.60	0.118
**HC without *Porites* spp.** ^{qb}				
Intercept	-2.98	0.56	-5.28	**<0.001**
Ω_{Ar}	0.52	0.18	2.91	**0.005**
**Complex HC** ^{qb}				
Intercept	-5.02	0.71	-7.08	**<0.001**
Ω_{Ar}	0.88	0.22	4.06	**<0.001**
**Non-complex HC** ^{qb}				
Intercept	-0.62	0.39	-1.60	0.118
Ω_{Ar}	-0.12	0.13	-0.89	0.381
***Acropora* spp.** ^{qb}				
Intercept	-5.52	1.04	-5.32	**<0.001**
Ω_{Ar}	0.75	0.32	2.34	**0.025**
**Acroporidae** ^{qb}				
Intercept	-4.53	0.86	-5.25	**<0.001**

Ω_{Ar}	0.68	0.27	2.51	**0.016**
***Pocillopora* spp.** [qb]				
Intercept	-6.91	1.29	-5.37	**<0.001**
Ω_{Ar}	0.95	0.39	2.42	**0.021**
***Seriatopora* spp.** [qb]				
Intercept	-8.61	1.61	-5.36	**<0.001**
Ω_{Ar}	1.40	0.48	2.93	**0.006**
**Pocilloporidae** [qb]				
Intercept	-6.98	0.98	-7.11	**<0.001**
Ω_{Ar}	1.16	0.29	3.93	**<0.001**
***Goniastrea* spp.** [qb]				
Intercept	-6.75	0.98	-6.91	**<0.001**
Ω_{Ar}	0.85	0.30	2.84	**0.007**
**Merulinidae** [qb]				
Intercept	-4.63	0.85	-5.46	**<0.001**
Ω_{Ar}	0.54	0.27	2.02	**0.050**
**Fungidae** [qb]				
Intercept	-8.92	1.43	-6.25	**<0.001**
Ω_{Ar}	1.20	0.43	2.81	**0.008**
**SC** [qb]				
Intercept	-4.89	1.11	-4.38	**<0.001**
Ω_{Ar}	0.19	0.36	0.52	0.604
**Sponges** [qb]				
Intercept	-2.38	0.52	-4.58	**<0.001**
Ω_{Ar}	-0.48	0.19	-2.52	**0.016**
**Calcareous algae** [qb]				
Intercept	-3.73	0.65	-5.74	**<0.001**
Ω_{Ar}	0.49	0.21	2.37	**0.023**
**CCA** [qb]				

Intercept	-7.84	1.03	-7.65	**<0.001**
Ω_{Ar}	1.16	0.31	3.77	**<0.001**
**Articulate calcareous Rhodophyta** [qb]				
Intercept	-9.37	1.47	-6.37	**<0.001**
Ω_{Ar}	1.72	0.43	4.00	**<0.001**
***Peyssonnelia* spp.** [qb]				
Intercept	-3.10	0.80	-3.88	**<0.001**
Ω_{Ar}	0.08	0.26	0.31	0.759
**Non-calcareous macroalgae** [qb]				
Intercept	-0.61	0.37	-1.67	0.104
Ω_{Ar}	-0.56	0.13	-4.19	**<0.001**
**Non-calcareous Phaeophyta** [qb]				
Intercept	-1.13	0.55	-2.03	**0.049**
Ω_{Ar}	-0.54	0.20	-2.70	**0.011**
**Non-calcareous Rhodophyta** [qb]				
Intercept	-1.98	0.67	-2.94	**0.006**
Ω_{Ar}	-0.63	0.26	-2.46	**0.019**
**Turf algae** [qb]				
Intercept	-0.50	0.30	-1.66	0.106
Ω_{Ar}	-0.10	0.10	-0.95	0.348
**Quadrat complexity** [qp]				
Intercept	-0.12	0.15	-0.83	0.413
Ω_{Ar}	0.35	0.05	7.712	**<0.001**
**Coral diversity and density**				
**HC adult diversity** [qp]				
Intercept	0.89	0.21	4.14	**<0.001**
Ω_{Ar}	0.42	0.07	6.22	**<0.001**
**HC juvenile diversity** [qp]				

Intercept	-0.22	0.29	-0.75	0.459
Ω_{Ar}	0.53	0.09	5.91	**<0.001**
**SC juvenile diversity** ^{qp}				
Intercept	-5.00	1.46	-3.42	**0.002**
Ω_{Ar}	1.18	0.44	2.70	**0.011**
**HC juvenile density** ^{qp}				
Intercept	0.13	0.38	0.34	0.736
Ω_{Ar}	0.64	0.12	5.51	**<0.001**
**SC juvenile density** ^{qp}				
Intercept	-5.87	1.82	-3.23	**0.003**
Ω_{Ar}	1.49	0.53	2.78	**0.009**
**Macroalgal biomass**				
**Total** $^{0.25\ g}$				
Intercept	2.16	0.28	7.63	**<0.001**
Ω_{Ar}	0.29	0.09	3.12	**0.004**
**Calcareous algae** $^{0.25\ g}$				
Intercept	-0.17	0.38	-0.46	0.650
Ω_{Ar}	0.82	0.12	6.61	**<0.001**
**Non-calcareous algae** $^{0.25\ g}$				
Intercept	2.61	0.27	9.55	**<0.001**
Ω_{Ar}	-0.02	0.09	-0.23	0.818
Turbinaria **spp.** $^{0.25\ g}$				
Intercept	-0.35	0.41	-0.86	0.394
Ω_{Ar}	0.73	0.13	5.40	**<0.001**
**Non-calcareous algae without** ***Turbinaria*** **spp.** $^{0.25\ g}$				
Intercept	3.31	0.31	10.71	**<0.001**
Ω_{Ar}	-0.43	0.10	-4.17	**<0.001**
Melanamansia **spp.** $^{0.25\ g}$				

Intercept	2.59	0.45	5.80	**<0.001**
Ω_{Ar}	-0.42	0.15	-2.86	**0.007**
**Calcareous algae : total algal biomass** **qb**				
Intercept	-4.64	0.88	-5.24	**<0.001**
Ω_{Ar}	1.37	0.28	4.96	**<0.001**

Supplementary Table 3: Genus level redundancy analysis (RDA) vector scores for the two axes explaining the most variation in benthic communities at the study stations. The vector codes are shown in Fig. 2.

Vector	RDA1	RDA2
Acropora spp.	0.356006	-0.32717
Astrea spp.	0.211185	-0.15727
Astreopora spp.	0.228424	-0.38562
Briareum spp.	-0.01966	0.178748
Calcareous rhodophyta (Calc Red)	0.532681	-0.19805
Coeloseris spp.	0.244107	0.033021
Crustose coralline algae (CCA)	0.517387	-0.17616
Ctenactis spp.	0.170641	0.120309
Cyanobacteria	0.155333	-0.5424
Cyphastrea spp.	0.062629	0.193453
Diploastrea spp.	0.121798	0.239334
Dpsastrea spp.	0.136892	0.207936
Echinopora spp.	0.130634	0.198091
Euphyllis spp.	0.155333	0.002932
Favites spp.	0.102934	0.222075
Fungia spp.	0.387116	-0.12115
Galaxea spp.	-0.18345	0.003644
Goniastrea spp.	0.418001	0.08397
Goniopora spp.	-0.00495	0.08941
Halimeda spp.	0.161062	-0.40903
Herpolitha spp.	0.063021	-0.53205
Hydnophora spp.	0.211018	-0.56659
Isopora spp.	0.257857	0.146616
Leptastrea spp.	0.094348	-0.43911
Leptoria spp.	0.23289	-0.03956
Lobophyllia spp.	-0.26144	-0.04549
Lobophytum spp.	-0.05506	-0.0376
Merulina spp.	0.115636	0.06465
Milepora	0.281301	-0.38267

Montipora spp.	0.235419	-0.26445
Nepthea spp.	0.262514	0.013134
Non-calcareous Chlorophyta	-0.03932	0.308528
Non-calcareous Phaeophyta (Ncalc Brown)	-0.32475	-0.27486
Non-calcareous Rhodophyta (Ncalc Red)	-0.35833	0.378124
Padina spp.	0.10612	-0.65048
Pavona spp.	-0.05333	-0.05753
Pectinia spp.	0.117433	-0.06182
Peyssonnelia spp.	0.04865	0.507137
Platygyra spp.	-0.16847	-0.10586
Pocillopora spp.	0.367232	-0.12621
Porites spp.	-0.23302	0.291484
Sarcophyton spp.	-0.43403	-0.13668
Seriatopora spp.	0.383562	-0.20349
Sinularia spp.	0.232704	0.039861
Sponge	-0.34279	-0.29129
Stylophora spp.	0.159356	-0.02529
Tubipora spp.	0.155333	0.002932
Turbinaria spp.	0.155101	0.129451
Turf algae	-0.07469	-0.13997
