# Supplementary material for: Progressive changes in coral reef communities with increasing ocean acidification
Source: Commun Biol. 2025 Nov 24;8:1518. doi: 10.1038/s42003-025-08889-w (PMC12644485; doi:10.1038/s42003-025-08889-w)
Supplement: Supplementary file 3 — Description of Additional Supplementary Files [file 42003_2025_8889_MOESM3_ESM.pdf]

Description of Additional Supplementary files

Supplementary Data 1: Carbon chemistry and algal biomass data from the monitoring station quadrats.

Supplementary Data 2: Carbon chemistry and benthic percent cover for different taxa from the monitoring station quadrats.

Supplementary Data 3: Carbon chemistry and adult coral diversity data from the monitoring station quadrats. The diversity data was derived from the point-intercept photographic surveys.

Supplementary Data 4: Carbon chemistry and juvenile hard and soft coral diversity and density data from the monitoring station quadrats.
