# Supplementary material for: Progressive changes in coral reef communities with increasing ocean acidification
Source: Commun Biol. 2025 Nov 24;8:1518. doi: 10.1038/s42003-025-08889-w (PMC12644485; doi:10.1038/s42003-025-08889-w)
Supplement: Supplementary file 8 — Reporting summary [file 42003_2025_8889_MOESM8_ESM.pdf]

nature portfolio

Corresponding author(s): Sam Noonan

Last updated by author(s): Jun 18, 2025

Reporting Summary

Nature Portfolio wishes to improve the reproducibility of the work that we publish. This form provides structure for consistency and transparency in reporting. For further information on Nature Portfolio policies, see our Editorial Policies and the Editorial Policy Checklist.

Statistics

For all statistical analyses, confirm that the following items are present in the figure legend, table legend, main text, or Methods section.

n/a	Confirmed	
☐	☒	The exact sample size (*n*) for each experimental group/condition, given as a discrete number and unit of measurement
☐	☒	A statement on whether measurements were taken from distinct samples or whether the same sample was measured repeatedly
☐	☒	The statistical test(s) used AND whether they are one- or two-sided<br>*Only common tests should be described solely by name; describe more complex techniques in the Methods section.*
☐	☒	A description of all covariates tested
☐	☒	A description of any assumptions or corrections, such as tests of normality and adjustment for multiple comparisons
☐	☒	A full description of the statistical parameters including central tendency (e.g. means) or other basic estimates (e.g. regression coefficient) AND variation (e.g. standard deviation) or associated estimates of uncertainty (e.g. confidence intervals)
☐	☒	For null hypothesis testing, the test statistic (e.g. *F*, *t*, *r*) with confidence intervals, effect sizes, degrees of freedom and *P* value noted<br>*Give P values as exact values whenever suitable.*
☒	☐	For Bayesian analysis, information on the choice of priors and Markov chain Monte Carlo settings
☐	☒	For hierarchical and complex designs, identification of the appropriate level for tests and full reporting of outcomes
☒	☐	Estimates of effect sizes (e.g. Cohen's *d*, Pearson's *r*), indicating how they were calculated

Our web collection on statistics for biologists contains articles on many of the points above.

Software and code

Policy information about availability of computer code

Data collection	Na
Data analysis	All statistical analyses were conducted in R (version 4.4.0) using a range of freely available packages. See "Statistics and reproducibility" section in the manuscript methods for R software and package details. Specific code for analyses is available upon request.

For manuscripts utilizing custom algorithms or software that are central to the research but not yet described in published literature, software must be made available to editors and reviewers. We strongly encourage code deposition in a community repository (e.g. GitHub). See the Nature Portfolio guidelines for submitting code & software for further information.

Data

Policy information about availability of data

All manuscripts must include a data availability statement. This statement should provide the following information, where applicable:

- Accession codes, unique identifiers, or web links for publicly available datasets
- A description of any restrictions on data availability
- For clinical datasets or third party data, please ensure that the statement adheres to our policy

All data from this publication are freely available via the AIMS data repository: https://apps.aims.gov.au/metadata/view/76c40b3c-8535-43b6-bf5f-ad26d6e1ad92

Research involving human participants, their data, or biological material

Policy information about studies with human participants or human data. See also policy information about sex, gender (identity/presentation), and sexual orientation and race, ethnicity and racism.

Reporting on sex and gender	Na
Reporting on race, ethnicity, or other socially relevant groupings	Na
Population characteristics	Na
Recruitment	Na
Ethics oversight	Na

Note that full information on the approval of the study protocol must also be provided in the manuscript.

Field-specific reporting

Please select the one below that is the best fit for your research. If you are not sure, read the appropriate sections before making your selection.

☐ Life sciences ☐ Behavioural & social sciences ☒ Ecological, evolutionary & environmental sciences

For a reference copy of the document with all sections, see nature.com/documents/nr-reporting-summary-flat.pdf

Ecological, evolutionary & environmental sciences study design

All studies must disclose on these points even when the disclosure is negative.

Study description	We examined changes in tropical coral reef benthic communities at 37 stations with varying exposure to submarine volcanic CO2 seeps, and determined the aragonite saturation state (ΩAr) where significant changes occur in situ.
Research sample	Research samples included:<br>- Seawater at each station to determine carbon chemistry parameters (e.g. pH and total alkalinity).<br>- Photographs of the seafloor at each station to examine benthic communities.<br>- Benthic algae communities at each station to examine algal biomass changes.
Sampling strategy	*Sampling stations were setup to capture a gradient of CO2 exposure. This was initially done visually before seawater carbon chemistry was characterised. Stations were established in areas surrounded by dense streams of CO2 bubbles, and spread well beyond the area of visible seeping, up to approximately 500 m to the south and north of the seep along the same island fringing reef. Hence stations were spread widely across and along the seep seascape, to capture varying seep intensities.*
Data collection	*-Seawater: bottle samples were collected ~twice-daily for two weeks at each station for pH and total alkalinity. SN and KF collected samples on SCUBA or snorkel. pH data was also collected using pH loggers.*<br>*-Photographs: SN and KF took benthic photos of a series of 1m2 quadrats at each station using a Canon camera and underwater housing.*<br>*-Algal samples: All macroalgae occurring within one quarter (i.e. 0.25 m2) of two of the quadrats per station were hand-collected by SN and KF using a scraping tool.*
Timing and spatial scale	*All data were collected during an expedition to Papua New Guinea in September - October 2016.*
Data exclusions	No data were excluded from the analyses.
Reproducibility	*Given the remote location of the unique study site, attempts have not been made to reproduce the study.*
Randomization	*Stations locations were haphazardly selected within the seep and along the adjacent fringing reef. Replicate quadrats were placed immediately around each station marker float, and all data were averaged between the quadrats prior to analyses to avoid any pseudo-replication.*
Blinding	*Blinding was used during the analysis of benthic community photographs. All were conducted by a single observer that was unaware of which station the photographs came from.*

Did the study involve field work? ☒ Yes ☐ No

Field work, collection and transport

Field conditions	*Field conditions were favorable during the sampling expedition. Both sea and air temperature were approximately 28 - 30 C. Water visibility was approximately 15m. Wave hight did not exceed 50cm.*
Location	*This study was conducted at the volcanic CO2 seep at Upa-Upasina, Normanby Island, Milne Bay, Papua New Guinea (PNG). Located 9.8192° S, 150.8147°E)*
Access & import/export	*This research was conducted under permits issued to Dr Katharina Fabricius from Papua New Guinea's Department of Environment and Conservation, and the National Research Institute. Permission was further gained from the family groups that inhabit the adjacent island and are the traditional custodians of the reefs studied.*
Disturbance	*The study was largely observational and sampling disturbance was minimal. We extracted some small water samples (50ml) for carbon chemistry analyses and removed algae samples from each station for biomass estimates. The sampling of benthic animals was limited to photography only.*

Reporting for specific materials, systems and methods

We require information from authors about some types of materials, experimental systems and methods used in many studies. Here, indicate whether each material, system or method listed is relevant to your study. If you are not sure if a list item applies to your research, read the appropriate section before selecting a response.

Materials & experimental systems

n/a	Involved in the study	
☒	☐	Antibodies
☒	☐	Eukaryotic cell lines
☒	☐	Palaeontology and archaeology
☐	☒	Animals and other organisms
☒	☐	Clinical data
☒	☐	Dual use research of concern
☐	☒	Plants

Methods

n/a	Involved in the study	
☒	☐	ChIP-seq
☒	☐	Flow cytometry
☒	☐	MRI-based neuroimaging

Antibodies

Antibodies used	*Na*
Validation	*Na*

Eukaryotic cell lines

Policy information about cell lines and Sex and Gender in Research

Cell line source(s)	*Na*
Authentication	*Na*
Mycoplasma contamination	*Na*
Commonly misidentified lines (See ICLAC register)	*Na*

Palaeontology and Archaeology

Specimen provenance	*Na*
Specimen deposition	*Na*
Dating methods	*Na*

☐ Tick this box to confirm that the raw and calibrated dates are available in the paper or in Supplementary Information.

Ethics oversight	*Na*

Note that full information on the approval of the study protocol must also be provided in the manuscript.

Animals and other research organisms

Policy information about studies involving animals; ARRIVE guidelines recommended for reporting animal research, and Sex and Gender in Research

Laboratory animals	*This study did not involve laboratory animals.*
Wild animals	*This study photographed wild corals and other benthic animals (eg clams, sponges etc). These animals were not caught or sampled in any other way.*
Reporting on sex	*Na*
Field-collected samples	*This study did not involve collected animal samples from the field.*
Ethics oversight	*No animal ethics oversight was required for this study as is was non-extractive and focused on invertebrates only (e.g. corals, sponges, clams etc).*

Note that full information on the approval of the study protocol must also be provided in the manuscript.

Clinical data

Policy information about clinical studies

All manuscripts should comply with the ICMJE guidelines for publication of clinical research and a completed CONSORT checklist must be included with all submissions.

Clinical trial registration	*Na*
Study protocol	*Na*
Data collection	*Na*
Outcomes	*Na*

Dual use research of concern

Policy information about dual use research of concern

Hazards

Could the accidental, deliberate or reckless misuse of agents or technologies generated in the work, or the application of information presented in the manuscript, pose a threat to:

No	Yes	
☒	☐	Public health
☒	☐	National security
☒	☐	Crops and/or livestock
☒	☐	Ecosystems
☒	☐	Any other significant area

Experiments of concern

Does the work involve any of these experiments of concern:

No	Yes	
☒	☐	Demonstrate how to render a vaccine ineffective
☒	☐	Confer resistance to therapeutically useful antibiotics or antiviral agents
☒	☐	Enhance the virulence of a pathogen or render a nonpathogen virulent
☒	☐	Increase transmissibility of a pathogen
☒	☐	Alter the host range of a pathogen
☒	☐	Enable evasion of diagnostic/detection modalities
☒	☐	Enable the weaponization of a biological agent or toxin
☒	☐	Any other potentially harmful combination of experiments and agents

Plants

Seed stocks: *Na*

Novel plant genotypes: *Na*

Authentication: *Na*

ChIP-seq

Data deposition

- [ ] Confirm that both raw and final processed data have been deposited in a public database such as GEO.
- [ ] Confirm that you have deposited or provided access to graph files (e.g. BED files) for the called peaks.

Data access links
May remain private before publication.: *Na*

Files in database submission: *Na*

Genome browser session (e.g. UCSC): *Na*

Methodology

Replicates: *Na*

Sequencing depth: *Na*

Antibodies: *Na*

Peak calling parameters: *Na*

Data quality: *Na*

Software: *Na*

Flow Cytometry

Plots

Confirm that:

- [ ] The axis labels state the marker and fluorochrome used (e.g. CD4-FITC).
- [ ] The axis scales are clearly visible. Include numbers along axes only for bottom left plot of group (a 'group' is an analysis of identical markers).
- [ ] All plots are contour plots with outliers or pseudocolor plots.
- [ ] A numerical value for number of cells or percentage (with statistics) is provided.

Methodology

Sample preparation: *Na*

Instrument: *Na*

Software: *Na*

Cell population abundance: *Na*

Gating strategy | Na

☐ Tick this box to confirm that a figure exemplifying the gating strategy is provided in the Supplementary Information.

Magnetic resonance imaging

Experimental design

Design type | Na

Design specifications | Na

Behavioral performance measures | Na

Acquisition

Imaging type(s) | Na

Field strength | Na

Sequence & imaging parameters | Na

Area of acquisition | Na

Diffusion MRI ☐ Used ☐ Not used

Preprocessing

Preprocessing software | Na

Normalization | Na

Normalization template | Na

Noise and artifact removal | Na

Volume censoring | Na

Statistical modeling & inference

Model type and settings | Na

Effect(s) tested | Na

Specify type of analysis: ☐ Whole brain ☐ ROI-based ☐ Both

Statistic type for inference | Na

(See Eklund et al. 2016)

Correction | Na

Models & analysis

n/a	Involved in the study
☐	☐ Functional and/or effective connectivity
☐	☐ Graph analysis
☐	☐ Multivariate modeling or predictive analysis

Functional and/or effective connectivity | Na

Graph analysis | Na

Multivariate modeling and predictive analysis | Na
